# A comparison of the effectiveness of respondent‐driven and venue‐based sampling for identifying undiagnosed HIV infection among cisgender men who have sex with men and transgender women in Tijuana, Mexico

**DOI:** 10.1002/jia2.25688

**Published:** 2021-03-23

**Authors:** Heather A Pines, Shirley J Semple, Carlos Magis‐Rodríguez, Alicia Harvey‐Vera, Steffanie A Strathdee, Rudy Patrick, Gudelia Rangel, Thomas L Patterson

**Affiliations:** ^1^ Department of Medicine University of California San Diego CA USA; ^2^ Department of Family Medicine and Public Health University of California San Diego CA USA; ^3^ Department of Psychiatry University of California San Diego CA USA; ^4^ Universidad Nacional Autónoma de Mexico Mexico City Mexico; ^5^ Universidad Xochicalco Tijuana Mexico; ^6^ United States‐Mexico Border Health Commission Tijuana Mexico; ^7^ El Colegio de la Frontera Norte Tijuana Mexico

**Keywords:** HIV testing, undiagnosed HIV infection, respondent‐driven sampling, venue‐based sampling, cisgender men who have sex with men, transgender women, Mexico

## Abstract

**Background:**

Efforts to increase HIV testing, diagnosis and care are critical to curbing HIV epidemics among cisgender men who have sex with men (MSM) and transgender women (TW) in low‐ and middle‐income countries (LMIC). We compared the effectiveness of respondent‐driven sampling (RDS) and venue‐based sampling (VBS) for identifying previously undiagnosed HIV infection among MSM and TW in Tijuana, Mexico.

**Methods:**

Between March 2015 and December 2018, we conducted RDS within the social networks of MSM and TW and VBS at venues frequented by MSM and TW to socialize and meet sexual partners. Those reached by RDS/VBS who reported at least 18 years of age, anal sex with MSM or TW, and no previous HIV diagnosis were eligible for HIV testing.

**Results:**

Of those screened following recruitment via RDS (N = 1232; 98.6% MSM; 1.3% TW), 60.8% (749/1232) were eligible for HIV testing and 97.5% (730/749) were tested for HIV infection, which led to the identification of 36 newly diagnosed HIV infections (4.9%). Of those screened following recruitment via VBS (N = 2560; 95.2% MSM; 4.6% TW), 56.5% (1446/2560) were eligible for HIV testing and 92.8% (1342/1446) were tested for HIV infection, which led to the identification of 82 newly diagnosed HIV infections (6.1%). The proportion of new HIV diagnoses did not differ by recruitment method (ratio = 0.81, 95% confidence interval: 0.55 to 1.18). Compared to those recruited via RDS, those tested following recruitment via VBS were younger, more likely to identify as gay, and more likely to identify as TW. Compared to those recruited via VBS, those newly diagnosed with HIV infection following recruitment via RDS reported higher levels of internalized stigma and were more likely to report injection drug use and a history of deportation from the United States.

**Conclusions:**

Despite RDS and VBS being equally effective for identifying undiagnosed HIV infection, each recruitment method reached different subgroups of MSM and TW in Tijuana. Our findings suggest that there may be benefits to using both RDS and VBS to increase the identification of previously undiagnosed HIV infection and ultimately support HIV care engagement among MSM and TW in Mexico and other similar LMIC.

## 1 Introduction

Key populations are often disproportionately affected by HIV in low‐ and middle‐income countries (LMIC) [1, 2], including cisgender men who have sex with men (MSM) and transgender women (TW) in Mexico whose HIV prevalence (17.3% and 17.4% respectively [3]) is approximately 85 times that of reproductive‐aged adults (0.2%) [4]. Tijuana is a large city along Mexico’s border with the United States (US) where the HIV risk environment is shaped by socio‐contextual factors (i.e. poverty, migration, substance use, sex work, stigma) that often intersect within key populations to facilitate HIV transmission and limit access to HIV services [5]. In a pooled analysis, only 11.5% of 191 persons living with HIV (PLWH) from key populations in Tijuana were previously diagnosed and <5% were receiving antiretroviral therapy (ART) [6]. Although universal access to ART has been available in Mexico since 2003 [7], strategies to overcome barriers posed by the HIV risk environment and facilitate HIV testing and entry into care are needed.

Despite World Health Organization (WHO) recommendations that MSM and TW undergo HIV testing at least annually [8], only 36.8% of those surveyed in Tijuana reported past year testing [9]. Among those never tested, barriers included fear of testing positive, low‐risk perception, confidentiality concerns, cost, mistrust of testing clinics, fear of discrimination from providers and the limited accessibility of testing clinics [9]. Efforts to engage MSM and TW in low cost, culturally competent HIV testing at conveniently located, non‐stigmatizing settings may be critical to increasing regular testing among MSM and TW in Tijuana.

Various recruitment methods have been used to engage key populations in HIV testing, including partner contract tracing (PCT), venue‐based sampling (VBS) and respondent‐driven sampling (RDS). PCT is an HIV/STI control strategy whereby newly diagnosed persons provide information on their recent sexual or injection partners so that they can be referred for testing [10, 11]. VBS and RDS were originally used in HIV surveillance and research to generate representative samples from marginalized populations and produce population HIV prevalence estimates but are now also used in HIV prevention. VBS identifies venues frequented by members of the target population and then recruits from those venues [12, 13]. However, VBS is limited to venues accessible to researchers and the persons who attend them. RDS overcomes that by enlisting and incentivizing members of the target population to recruit peers from their social networks who are then incentivized to recruit their peers [14, 15]. However, RDS samples can be biased as persons tend to recruit peers similar to themselves and those with larger networks are often overrepresented [16]. Moreover, the methods for addressing these biases may not yield valid population HIV prevalence estimates [17, 18]. Nevertheless, that does not hinder the use of VBS or RDS for the identification of undiagnosed HIV [19, 20], and strategies like RDS are recommended for HIV testing recruitment by the WHO [21] and Centers for Disease Control and Prevention [22].

While the relative effectiveness of these recruitment methods for identifying undiagnosed HIV remains understudied among MSM and TW in LMIC, it has varied across US‐based studies. No difference in the proportion of those newly diagnosed with HIV following PCT, VBS or RDS recruitment was observed in a Washington DC study [23], whereas a New York City study found that PCT and RDS were equivalent but more effective than VBS [24], which differs from a Baltimore study where VBS was more effective than both PCT and RDS [25]. In another New York City study, RDS was less effective at identifying newly or previously diagnosed HIV than VBS and online recruitment where those recruited also recruited peers [26]. Within these studies, the relative effectiveness of the methods compared was attributed to the varying subgroups of MSM and TW they each reached, which may reflect contextual factors that affect the extent to which they engage different subgroups across diverse settings [27, 28, 29].

*Proyecto Enlaces* (Links Project) was launched in March 2015 to compare the effectiveness of PCT and VBS for identifying undiagnosed HIV among MSM and TW in Tijuana. Due to implementation challenges (i.e. limited partner contact information, low acceptability partially due to mistrust of staff performing referrals) [30], RDS replaced PCT in March 2016. We (1) compared the effectiveness of VBS and RDS for identifying undiagnosed HIV among MSM and TW in Tijuana, (2) determined whether the characteristics of those tested and newly diagnosed differed by recruitment method and (3) examined the cost utility of each method.

## 2 Methods

*Proyecto Enlaces* was conducted between March 2015 and December 2018 out of a field site in Tijuana’s *Zona Norte* neighbourhood, which is adjacent to the Mexico‐US border, overlaps with Tijuana’s red‐light district, and is where many of the venues frequented by MSM and TW to socialize and meet sexual partners in Tijuana are located [31]. While the study’s primary goal was to compare the effectiveness of VBS and RDS for identifying undiagnosed HIV, its secondary goal was to examine the extent to which sexual, venue affiliation and inferred HIV transmission networks overlap among PLWH recruited via VBS and RDS at baseline and 12 months. Study procedures were performed by local staff with experience working with sexual and gender minorities. Staff were trained to implement VBS and RDS and provide culturally competent HIV counselling and testing services. Based on pilot data (20% HIV prevalence, 89% unaware of their HIV‐positive status among MSM and TW recruited via RDS) [32], we aimed to recruit approximately 1250 persons through VBS and 1000 persons through RDS for HIV testing in order to identify 200 newly diagnosed persons via each method. Participants provided informed consent and study procedures were approved by ethics committees at the University of California, San Diego and Xochicalco University.

### 2.1 Venue‐based sampling

As described [33, 34, 35], VBS was conducted using time‐location sampling methods [12]. Through formative research and information from participants, we identified venues frequented by MSM and TW in Tijuana, which included 34 physical venues (e.g. bars/clubs, public spaces), one virtual venue (i.e. sex‐seeking geo‐spatial social networking [GSN] application) and six special events (e.g. Gay Pride). Prior to initiating VBS at a venue, staff obtained permission from venue management (if applicable) to conduct on‐site recruitment and determined days and times MSM and TW attend the venue to generate our sampling frame of four‐hour venue‐day‐time slots. Each month, staff pre‐selected venue‐day‐time slots (approximately five days per week) in which to conduct VBS, including primary and back‐up venues in case a venue was closed or had fewer patrons than expected. At venues, staff approached persons to tell them about the study and conduct eligibility screening. To incentivize screening, persons were invited to spin a wheel that offered non‐coercive prizes (e.g. flavoured lubricant). Eligible persons were offered HIV testing on‐site or scheduled at the study site if preferred. Eligibility criteria for HIV testing included: ≥18 years old, cisgender male or transgender female, anal sex with a cisgender male or transgender female in the past four months, no previous HIV diagnosis and willing to provide oral (on‐site) or written (study site) informed consent. Persons reporting a previous HIV diagnosis were eligible for other aims.

### 2.2 Respondent‐driven sampling

As described [33, 34, 35], RDS was initiated by 33 seeds (33% enrolled within the first month) identified through VBS or referrals from organizations that serve PLWH and selected throughout the study period to enhance diversity with respect to HIV status, age, socioeconomic status, sexual orientation, gender identity and recruitment source. Eligibility criteria for seeds included: ≥18 years old, cisgender male or transgender female, anal sex with a cisgender male or transgender female in the past four months, and willing to provide written informed consent. To minimize the potential for unproductive seeds, we excluded persons residing outside Tijuana and persons with <15 MSM or TW residents in their social networks (<5 beginning approximately 12 months into RDS to boost recruitment). To measure social network size, potential seeds were asked how many MSM and TW they know who are at least 18 years old and live in Tijuana and, of those, how many know them back [32]. Seeds received three coupons (six beginning approximately 22 months into RDS to boost recruitment), which they used to refer MSM or TW from their social networks to the study. Eligible peer recruits who presented with a coupon also received three to six coupons to refer MSM or TW from their social networks. Eligibility criteria for peer recruits to refer peers included: ≥18 years old, cisgender male or transgender female, anal sex with a cisgender male or transgender female in the past 12 months (so as not to prematurely terminate recruitment chains, but only those reporting anal sex in the past four months contributed to the present analysis for comparability with VBS), and willing to provide written informed consent. Peer recruits who did not report a previous HIV diagnosis were eligible for HIV testing at the study site, whereas those who did were eligible for other aims. Staff helped participants develop peer outreach plans and participants received $100 Mexican pesos (approximately US $5) per peer referred.

### 2.3 HIV testing and HIV diagnosis

Eligible MSM and TW underwent rapid HIV testing (Advanced Quality HIV 1/2 Test Kits, Intec Products Inc). Persons recruited multiple times via VBS or RDS who remained eligible for testing were re‐tested if it had been at least three months since their last test. Rapid test results, post‐test counselling and incentives of $150 Mexican pesos (approximately US $8) were delivered within a few days at the study site to those tested at a venue (to protect participant and staff safety) or immediately to those tested at the study site. Persons with positive rapid test results were offered enrolment in a 12‐month study, provided a blood sample for confirmatory HIV testing (Bio‐Rad Geenius® HIV 1/2 Confirmatory Assay and chemiluminescence assay) at the San Diego County Public Health Laboratory (SDCPHL), and consented to HIV care data abstraction from their medical records at Tijuana’s federally funded HIV clinic (CAPASITS) where key populations are most likely to access care. Within two weeks, participants received confirmatory test results and referrals to CAPASITS for free HIV care. Those confirmed positive without evidence of previous HIV care at CAPASITS were considered newly diagnosed.

### 2.4 Data collection

Eligibility screeners collected age, gender identity, sexual orientation, anal sex with MSM or TW in the past four and 12 months, and previous HIV diagnosis. Social network size was also ascertained from RDS participants. Newly diagnosed participants enrolled in the 12‐month study completed interviewer‐administered surveys to collect: sociodemographics (education; employment; average monthly income; duration of residence in Tijuana), history of incarceration and deportation from the US, psychosocial factors (social support [36]; internalized stigma [37] related to having sex with men [MSM only] or gender identity [TW only]; outness [38] about having sex with men [MSM only] or being a transgender woman [TW only]; depressive symptoms [39]), HIV knowledge [40], HIV testing history, hazardous alcohol consumption [41], illicit drug use (past month) and data on up to 20 anal or vaginal sex partners (past four months) via an egocentric sexual network inventory [42].

### 2.5 Statistical analysis

We compared the proportion of persons newly diagnosed with HIV (primary outcome), engaged with the study (secondary outcomes; eligible for testing, tested) and newly or previously diagnosed with HIV (secondary outcome) by recruitment method using Poisson regression with a robust error variance. Chi‐square, Fisher’s exact and Wilcoxon rank sum tests were used to compare characteristics of participants tested and newly diagnosed by recruitment method. Seeds did not contribute to the RDS sample in these analyses. Although eligible persons recruited multiple times were re‐tested for HIV, analyses were restricted to participants’ initial recruitment date. Analyses were performed using SAS 9.4 (SAS Institute, Inc. Cary, North Carolina, USA). Consistent with prior studies that compared the effectiveness of VBS and RDS for identifying undiagnosed HIV [23, 24, 25, 26], we did not apply VBS or RDS sampling weights in our analyses. Since our goal was not to obtain population estimates, but instead to directly compare participants recruited via each method, data needed to construct VBS sampling weights were not collected. However, RDS Analysis Tool (Version 7.1.46) was used to generate population estimates for the RDS sample.

### 2.6 Cost‐utility analysis

We compiled the following observed costs (in 2017 US dollars) associated with VBS and RDS throughout their planning and implementation: (1) personnel, (2) GSN advertisements, (3) equipment, (4) materials and supplies, (5) incentives, (6) transportation and (7) overhead. We divided the total cost of implementing each recruitment method by (1) the number of persons recruited via each method tested for HIV to estimate the cost per test and (2) the number of persons recruited via each method newly diagnosed with HIV to estimate the cost per diagnosis.

## 3 Results

### 3.1 VBS sample

Over 46 months, VBS led to the recruitment of 2677 persons for screening across 1071 venue‐day‐time slots: 86% led to ≥1 person screened (mean = 3.6 per slot; standard deviation [SD] = 4.0), 80% led to ≥1 person eligible for HIV testing (mean = 2.5 per slot, SD = 2.4) and 77% led to ≥1 person tested (mean = 2.3 per slot; SD = 2.4). Overall, 80% of persons tested at a venue visited the study site for their results.

**Figure 1:**
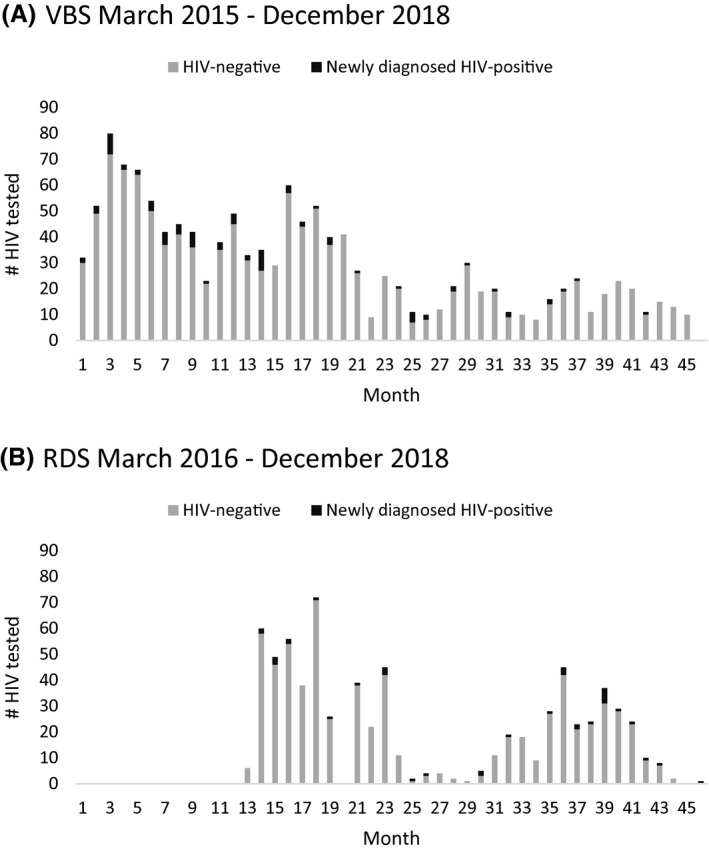
Number of participants HIV tested and newly diagnosed with HIV infection by month following recruitment via **(A)** venue‐based sampling (VBS) and **(B)** respondent‐driven sampling (RDS) in Tijuana, Mexico.

Of the 2560 persons initially screened following VBS recruitment (Table 1; Table S1), 56.5% were eligible for HIV testing, of whom 92.8% were tested, with an average of 29.2 (SD = 18.4) persons tested per month (Figure 1A). Of those tested, 6.1% were newly diagnosed, with a greater proportion of new diagnoses occurring among TW (12.8%) than MSM (5.7%) (*p* = 0.02). While not statistically significant, the proportion of new diagnoses varied across venue types (Table S2), and was highest at virtual venues (11.1%), bathhouses (9.9%) and bars/clubs (6.5%). Overall, 9.6% of those recruited via VBS were newly or previously diagnosed PLWH.

**Table 1:** Primary and secondary outcomes by recruitment method among MSM and TW in Tijuana, Mexico.

	RDS			VBS			
	TW	MSM	Total	TW	MSM	Total	Ratio^a^
	n (%)	n (%)	n (%)	n (%)	n (%)	n (%)	(95% CI)
Primary outcome							
Newly diagnosed HIV infection^b^	2 (16.7)	34 (4.7)	36 (4.9)	11 (12.8)	71 (5.7)	82 (6.1)	0.81 (0.55, 1.18)
Secondary outcomes							
Screened for eligibility	16	1215	1232	118	2438	2560	
Ineligible for HIV testing^c^	4 (25.0)	478 (39.3)	483 (39.2)	21 (17.8)	1086 (44.6)	1114 (43.5)	
≤18 years of age	0 (0.0)	0 (0.0)	0 (0.0)	3 (14.3)	7 (0.6)	11 (1.0)	
Cisgender female	0 (0.0)	0 (0.0)	1 (0.2)	0 (0.0)	0 (0.0)	7 (0.6)	
No anal sex (past 4 months)	2 (50.0)	457 (95.6)	459 (95.0)	14 (66.7)	1031 (94.9)	1046 (93.9)	
Previously diagnosed HIV infection	2 (50.0)	27 (5.7)	29 (6.0)	4 (19.0)	48 (4.4)	52 (4.7)	
Eligible for HIV testing^c^	12 (75.0)	737 (60.7)	749 (60.8)	97 (82.2)	1349 (55.4)	1446 (56.5)	1.08 (1.02, 1.14)
HIV tested^d^	12 (100.0)	718 (97.4)	730 (97.5)	86 (88.7)	1256 (93.1)	1342 (92.8)	1.05 (1.03, 1.07)
Any HIV infection^e^	4 (28.6)	61 (8.2)	65 (8.6)	15 (16.7)	119 (9.1)	134 (9.6)	0.89 (0.67, 1.18)

CI, confidence interval; MSM, cisgender men who have sex with men; RDS, respondent‐driven sampling; TW, transgender women; VBS, venue‐based sampling.

^a^ Ratio of the proportion of the RDS sample with the outcome to the proportion of the VBS sample with the outcome

^b^ Among tested

^c^ Among screened

^d^ Among eligible

^e^ Newly or previously diagnosed, among tested and previously diagnosed.

### 3.2 RDS sample

Over 34 months, 33 seeds initiated RDS (mean age: 31.9 years; 85% MSM; 79% gay‐identifying; 62% ≥high school education; 27% HIV‐positive; 88% identified via VBS) and led to the recruitment of 1279 peer recruits for screening across 18 recruitment chains, 73% (N = 934) of whom were eligible to recruit peers (Figure S1; Table S3). Half of seeds (55%; mean = 2.3 peers recruited per seed) and eligible peer recruits (50%; mean = 0.96 peers recruited per peer recruit) recruited ≥ 1 eligible peer.

Of the 1232 peer recruits initially screened following RDS recruitment (Table 1; Table S1), 60.8% were eligible for HIV testing, of whom 97.5% were tested, with an average of 21.5 (SD = 19.4) peer recruits tested per month (Figure 1B). Of those tested, 4.9% were newly diagnosed (61% reported no VBS venue attendance; Table S4), with a greater proportion of new diagnoses occurring among TW (16.7%) than MSM (4.7%) (*p* = 0.11) and after participants started receiving six coupons (7.8% vs 3.6%; *p* = 0.02). Overall, 8.6% of those recruited via RDS were newly or previously diagnosed PLWH.

### 3.3 Primary and secondary outcomes by recruitment method

Neither the proportion eligible for testing, tested, newly diagnosed, nor living with HIV differed meaningfully by recruitment method (Table 1).

### 3.4 Characteristics of participants tested and newly diagnosed by recruitment method

Compared to those tested following RDS recruitment (Table 2), those recruited via VBS were younger (median age: 32.0 vs 42.0 years) and more often identified as TW (6.4% vs 1.6%). Nearly equal proportions of participants tested following VBS recruitment identified as gay (39.6%) and bisexual (40.8%), whereas a greater proportion of those recruited via RDS identified as bisexual (58.4%). Participants newly diagnosed following RDS recruitment were older (median age: 37.0 vs 30.0 years) and reported greater levels of internalized stigma (median stigma score: 25.5 vs 19.0) (Table 3). Although not statistically significant, a greater proportion of those newly diagnosed following VBS recruitment reported identifying as TW (13.4% vs 5.6%), being employed (71.3% vs 52.9%) and hazardous alcohol consumption (40.0% vs 20.6%), whereas a greater proportion of those newly diagnosed following RDS recruitment reported deportation from the US (39.4% vs 26.3%) and injection drug use (17.7% vs 8.8%). No meaningful or statistically significant differences in sexual behaviours linked to HIV, HIV knowledge or HIV testing were observed between those newly diagnosed by method.

**Table 2:** Characteristics of MSM and TW tested for HIV infection by recruitment method

Characteristic	RDS (N = 730)		VBS (N = 1342)		*p*‐value
	n	%	n	%	
Median age (years)	42.0	IQR = 31.0 to 49.0	32.0	IQR = 25.0 to 42.0	<0.0001
Age (years)					
18 to 24	83	11.4	326	24.3	<0.0001
25 to 29	65	8.9	244	18.2	
30 to 39	162	22.2	375	27.9	
40 to 49	243	33.3	264	19.7	
50+	177	24.3	133	9.9	
Gender identity					
Cisgender male	718	98.4	1256	93.6	<0.0001
Transgender female	12	1.6	86	6.4	
Median years of residence in Tijuana	7.0	IQR = 2.0 to 20.0	11.0	IQR = 3.0 to 23.0	0.0002
Sexual orientation					
Heterosexual	103	14.1	250	18.7	<0.0001
Bisexual	426	58.4	547	40.8	
Gay	182	25.0	530	39.6	
Other/not sure	18	2.5	13	1.0	

Numbers may not sum to column total due to missing data; percentages may not sum to 100 due to rounding or omission of one category for binary variables. IQR, interquartile range; MSM, cisgender men who have sex with men; RDS, respondent‐driven sampling; TW, transgender women; VBS, venue‐based sampling.

**Table 3:** Characteristics of MSM and TW newly diagnosed with HIV infection by recruitment method

	RDS (N = 36)		VBS (N = 82)		*p*‐value
	n	%	n	%	
Socio‐demographics					
Median age (years)	37.0	IQR = 28.0 to 44.0	30.0	IQR = 24.0 to 37.0	0.01
Age (years)					
18 to 24	7	19.4	23	28.1	0.06
25 to 29	3	8.3	17	20.7	
30 to 39	11	30.6	28	34.2	
40 to 49	11	30.6	10	12.2	
50+	4	11.1	4	4.9	
Gender identity					
Cisgender male	34	94.4	71	86.6	0.34
Transgender female	2	5.6	11	13.4	
Median years of residence in Tijuana	6.5	IQR = 2.0 to 15.0	10.0	IQR = 3.0 to 22.0	0.11
Sexual orientation					
Heterosexual	3	8.3	8	9.8	0.37
Bisexual	13	36.1	23	28.1	
Gay	19	52.8	51	62.2	
Other/not sure	1	2.8	0	0.0	
≥High school education	18	52.9	38	47.5	0.60
Employed	18	52.9	57	71.3	0.06
Average monthly income ≥$3000 MXN (approximately $150 USD)	0	0.0	0	0.0	–
History of incarceration	9	26.5	22	27.5	1.00
History of deportation from the US	13	39.4	21	26.3	0.17
Psychosocial factors					
Social support, median score (range: 0 to 100)	75.0	IQR = 46.9 to 96.9	65.6	IQR = 43.8 to 78.1	0.28
Internalized stigma, median score (range: 9 to 45)	25.5	IQR = 22.0 to 32.0	19.0	IQR = 14.0 to 28.0	0.01
Outness, median score (range: 1 to 7)	5.0	IQR = 1.0 to 7.0	5.0	IQR = 4.0 to 7.0	0.31
Depressive symptoms (CESD‐10 score ≥10)	15	44.1	29	36.7	0.46
Substance use behaviours					
Any illicit drug use (past month)^a^	15	44.1	30	37.5	0.51
Any injection drug use (past month)	6	17.7	7	8.8	0.20
Hazardous alcohol consumption (AUDIT score ≥7)	7	20.6	32	40.0	0.05
Sexual behaviours (past 4 months)					
Any alcohol or illicit drug use before or during sex^a^	26	76.5	62	77.5	1.00
Any condomless anal sex	28	82.4	63	78.8	0.80
Any exchange of something of value for sex	10	29.4	26	32.5	0.83
Sexual network characteristics (past 4 months)					
Median sexual network size	4.0	IQR = 2.0 to 8.0	3.0	IQR = 1.0 to 9.0	0.35
Any HIV‐positive/status unknown sexual partners	29	85.3	58	72.5	0.16
Any main or primary sexual partners	10	29.4	44	55.0	0.01
Any cisgender female sexual partners	10	29.4	10	12.5	0.06
Any transgender female sexual partners	4	11.8	3	3.8	0.19
HIV knowledge and prevention					
HIV knowledge, median score (range: 0 to 18)	16.0	IQR = 15.0 to 17.0	16.0	IQR = 15.0 to 17.0	0.95
Lifetime HIV testing^b^	17	68.0	37	68.5	1.00
HIV testing (past 12 months)^b^	9	36.0	17	31.5	0.80

Numbers may not sum to column total due to missing data; percentages may not sum to 100 due to rounding or omission of one category for binary variables. AUDIT, Alcohol Use Disorders Identification Test; CESD‐10, 10‐item Center for Epidemiologic Studies Depression Scale; IQR, interquartile range; MSM, cisgender men who have sex with men; MXN, Mexican pesos; RDS, respondent‐driven sampling; TW, transgender women; USD, United States dollar; VBS, venue‐based sampling.

^a^ Illicit drugs include methamphetamine, cocaine, heroin, inhalants, amyl nitrites (poppers), ecstasy, gamma‐hydroxybutyrate, ketamine, tranquilizers, or barbiturates

^b^ collected at 2‐week follow‐up visit, data missing for 39 participants who did not return for that visit

### 3.5 Cost utility

The costs per test and per diagnosis for RDS (US $260 and $5273) were slightly higher than those for VBS (US $234 and $3828) (Table 4).

**Table 4:** Costs in United States dollars associated with planning and implementing RDS (2016 to 2018) and VBS (2015 to 2018) for the identification of newly diagnosed HIV infection among MSM and TW in Tijuana, Mexico

Cost category	RDS	VBS
Personnel (e.g. staff salaries)	111,500	200,357
GSN advertisements	–	4500
Equipment (e.g. laptops, cell phone, VBS testing canopy, VBS prize wheel)	2107	2900
Materials/supplies (e.g. flyers, coupons, rapid and confirmatory HIV testing supplies)	7341	10,548
Incentives (e.g. testing incentive, VBS prizes, RDS peer recruitment incentive)	10,327	11,754
Transportation (e.g. staff to venues, blood sample transport to SDCPHL)	11,900	20,700
Overhead (e.g. rent and utilities)	46,648	63,112
Total cost	189,823	313,871
Cost per HIV test	260	234
Cost per newly diagnosed HIV infection	5273	3828

GSN, geo‐spatial social networking application; MSM, cisgender men who have sex with men; RDS, respondent‐driven sampling; SDCPHL, San Diego County Public Health Laboratory; TW, transgender women; VBS, venue‐based sampling.

## 4 Discussion

We compared the effectiveness and costs of VBS and RDS for identifying undiagnosed HIV among MSM and TW in Tijuana. While costs were slightly higher for RDS than VBS, the proportion of new HIV diagnoses did not differ by recruitment method. Prior work has similarly observed no difference in their effectiveness for identifying new diagnoses [23]; however, other studies have also documented differential levels of effectiveness, with the direction of their relative effectiveness varying across studies [24, 25, 26]. Our findings may be explained by the fact that sexual behaviours linked to HIV and HIV testing history did not differ by recruitment method.

Consistent with prior work that compared VBS and RDS for recruiting [27, 28, 29] and identifying undiagnosed HIV [23, 24, 25, 26], we found that each method reached different high‐risk subgroups. Compared to RDS, VBS engaged younger persons, both gay‐ and bisexual‐identifying persons, and more TW in testing. Among newly diagnosed participants, employment and hazardous alcohol consumption were reported by a greater proportion of those recruited via VBS, while internalized stigma related to sexual or gender identity, deportation and injection drug use were more common among those recruited via RDS. Observed differences between participants recruited via each method may be due to the fact that VBS, particularly when concentrated at bars and clubs, reaches MSM and TW who are more out about their sexual or gender identity and more connected to the LGBTQ community. Persons who inject drugs, deportees, and those more affected by stigma often experience social marginalization and healthcare exclusion in Tijuana [9, 43, 44, 45]. Thus, although 88% of seeds were selected from persons recruited via VBS, through successive recruitment waves, RDS appears to have leveraged the social networks of MSM and TW to reach these more marginalized subgroups, which may be harder to engage through VBS alone.

We also found that the proportion of PLWH did not differ by recruitment method and was lower than that in Tijuana pilot studies (2012 to 2013) conducted among MSM and TW recruited via RDS (unweighted = 17%; weighted = 20% [12.5% to 29.1%]) and TW recruited via VBS (unweighted = 22%) [32, 46], but was comparable to that among MSM recruited via VBS in Northwest Mexico in 2011 (weighted = 11.8%, [10.1% to 13.5%]) [47]. While our study was larger than the pilot studies, and likely more reflective of the broader population of MSM and TW in Tijuana given the number of waves in our longest RDS recruitment chains and the range of venues in which we conducted VBS, it remains unclear whether the proportion of PLWH among MSM and TW in Tijuana has decreased or remained roughly constant over time. In prior work with MSM and TW in Tijuana, fear of testing positive was the most common barrier to HIV testing [9]. Despite efforts to provide culturally competent HIV counselling and testing, this fear may have remained a barrier among those at greatest risk and contributed to our identification of fewer PLWH. However, consistent with the two pilot studies [32, 46], the majority of PLWH we reached by VBS and RDS were previously unaware of their HIV‐positive status.

Our study has several limitations. First, our use of self‐reported HIV status during eligibility screening may have led to the misclassification of some previously diagnosed persons as newly diagnosed, as noted in prior research [48, 49]. While we were able to prevent this to some degree by identifying those who had previously accessed HIV care at CAPASITS, their eligibility to contribute to other aims may have helped enhance the accuracy of self‐reports. Second, given our focus, we did not apply VBS or RDS sampling weights in our analyses, and therefore our findings may not be generalizable to all MSM and TW in Tijuana. Nevertheless, our findings do reflect the ability of VBS and RDS to engage diverse subgroups of MSM and TW in HIV testing to identify undiagnosed HIV in Tijuana, as well as the costs associated with their implementation for that purpose. Finally, while we were able to engage a large number of MSM and TW in HIV testing and detect meaningful differences between those recruited via VBS and RDS, the proportion of new diagnoses was lower than expected. Therefore, analyses among newly diagnosed participants were underpowered to detect statistically significant differences by recruitment method.

## 5 Conclusions

Studies have examined the relative effectiveness of VBS and RDS for identifying undiagnosed HIV among MSM and TW in the US; however, less is known with respect to their relative effectiveness in LMIC. Our findings highlight the potential for both VBS and RDS to facilitate HIV testing among MSM and TW in Tijuana. While these methods may be equally effective for identifying undiagnosed HIV in this setting, the diversity of those recruited via each method suggests there may be benefits to using both to ensure the identification of undiagnosed HIV among all MSM and TW at elevated risk. Recognizing the higher costs associated with RDS, the staff time required to conduct VBS, and that the cost per HIV diagnosis for both methods may exceed that considered cost‐effective [50], adapted strategies, such as the hybrid approach evaluated in New York City where those recruited via VBS also recruited peers [26], may be warranted to facilitate their combined implementation. Further adaptations could include targeting VBS to venues where the yield of new diagnoses was greatest (i.e. virtual venues, bathhouses, bars/clubs) and not requiring potential seeds to have overly large social networks or removing limits on the number of RDS coupons distributed, both of which proved too restrictive in our study. Future analyses will examine new diagnoses clustered in our RDS recruitment chains and ways that HIV testing could be further targeted among VBS and RDS recruits to maximize their yield.

## Competing Interest

The authors declare that they have no conflicts of interest.

## Authors’ Contributions

HAP, SJS, CMR, SAS and TLP contributed to the conception and design of the study. HAP, AHV and GR oversaw study procedures and data collection. HAP analysed the data, with assistance from RP, and drafted the manuscript. All authors contributed to the interpretation of results, manuscript revisions and read and approved the final manuscript.

## Supporting information


**Figure S1**. Respondent‐driven sampling (RDS) recruitment chains among 967 cisgender men who have sex with men (MSM) and transgender women (TW) in Tijuana, Mexico (33 seeds; 934 eligible peer recruits). Recruitment chains had a mean of 5.3 waves (standard deviation [SD]=7.4), with the two longest chains having 31 and 17 waves. Seeds and peer recruits had mean social network sizes of 51.6 (SD = 93.4) and 13.9 (SD = 34.2), respectively. Peer recruits most commonly reported recruitment by a friend (55%), acquaintance (35%), or sex partner (4%). All seeds and 85.6% of eligible peer recruits reported anal sex with cisgender men or TW in the past four months.
**Table S1**. MSM and TW in Tijuana, Mexico screened for HIV testing eligibility by initial recruitment method.
**Table S2**. MSM and TW in Tijuana, Mexico tested and newly diagnosed with HIV infection following VBS recruitment by venue and venue type.
**Table S3**. Cross‐group recruitment probabilities, homophily, and relevant proportions by age, gender identity, sexual orientation, sexual activity, and HIV status among MSM and TW recruited via RDS in Tijuana, Mexico.
**Table S4**. Characteristics of MSM and TW in Tijuana, Mexico newly diagnosed with HIV infection following recruitment via RDS by VBS recruitment venue attendance in the past four months.
