# Supplementary material for: A comparison of the effectiveness of respondent‐driven and venue‐based sampling for identifying undiagnosed HIV infection among cisgender men who have sex with men and transgender women in Tijuana, Mexico
Source: J Int AIDS Soc. 2021 Mar 23;24(3):e25688. doi: 10.1002/jia2.25688 (PMC7987819; doi:10.1002/jia2.25688)
Supplement: Supplementary file 1 — Figure S1. Respondent‐driven sampling (RDS) recruitment chains among 967 cisgender men who have sex with men (MSM) and transgender women (TW) in Tijuana, Mexico (33 seeds; 934 eligible peer recruits). Recruitment chains had a mean of 5.3 waves (standard deviation [SD]=7.4), with the two longest chains having 31 and 17 waves. Seeds and peer recruits had mean social network sizes of 51.6 (SD = 93.4) and 13.9 (SD = 34.2), respectively. Peer recruits most commonly reported recruitment by a friend (55%), acquaintance (35%), or sex partner (4%). All seeds and 85.6% of eligible peer recruits reported anal sex with cisgender men or TW in the past four months. Table S1. MSM and TW in Tijuana, Mexico screened for HIV testing eligibility by initial recruitment method. Table S2. MSM and TW in Tijuana, Mexico tested and newly diagnosed with HIV infection following VBS recruitment by venue and venue type. Table S3. Cross‐group recruitment probabilities, homophily, and relevant proportions by age, gender identity, sexual orientation, sexual activity, and HIV status among MSM and TW recruited via RDS in Tijuana, Mexico. Table S4. Characteristics of MSM and TW in Tijuana, Mexico newly diagnosed with HIV infection following recruitment via RDS by VBS recruitment venue attendance in the past four months. [file JIA2-24-e25688-s001.docx]

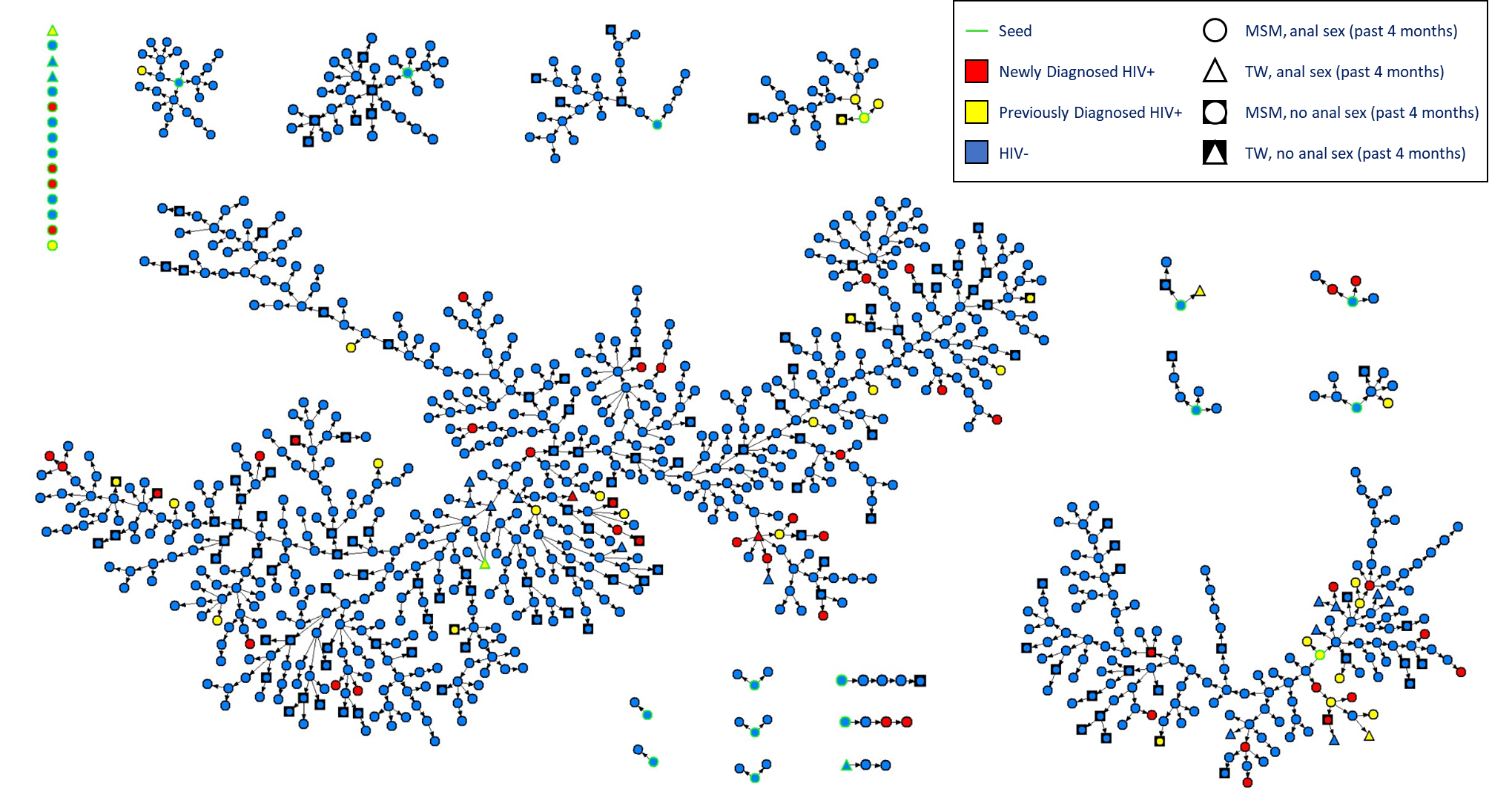


**Supplemental Figure 1.** Respondent-driven sampling (RDS) recruitment chains among 967 cisgender men who have sex with men (MSM) and transgender women (TW) in Tijuana, Mexico (33 seeds; 934 eligible peer recruits). Recruitment chains had a mean of 5.3 waves (standard deviation [SD]=7.4), with the two longest chains having 31 and 17 waves. Seeds and peer recruits had mean social network sizes of 51.6 (SD=93.4) and 13.9 (SD=34.2), respectively. Peer recruits most commonly reported recruitment by a friend (55%), acquaintance (35%), or sex partner (4%). All seeds and 85.6% of eligible peer recruits reported anal sex with cisgender men or TW in the past 4 months.

| **Supplemental Table 1. MSM and TW in Tijuana, Mexico screened for HIV testing eligibility by initial recruitment method.** | | | | | | | | | | |  |  |  |  |  |
| --- | --- | --- | --- | --- | --- | --- | --- | --- | --- | --- | --- | --- | --- | --- | --- |
|  | | **RDS (N=1232)** | | | | **VBS (N=2560)** | | | |  |  |  |  |  |  |
|  | | **n** | | **(%)** | | **n** | | **(%)** | |  |  |  |  |  |  |
| **Number of times screened** | |  | |  | |  | |  | |  |  |  |  |  |  |
| 1 | | 1024 | | 83.12 | | 2191 | | 85.59 | |  |  |  |  |  |  |
| 2 | | 147 | | 11.93 | | 167 | | 6.52 | |  |  |  |  |  |  |
| 3 | | 34 | | 2.76 | | 83 | | 3.24 | |  |  |  |  |  |  |
| 4 | | 7 | | 0.57 | | 37 | | 1.45 | |  |  |  |  |  |  |
| 5 | | 8 | | 0.65 | | 22 | | 0.86 | |  |  |  |  |  |  |
| 6 | | 4 | | 0.32 | | 12 | | 0.47 | |  |  |  |  |  |  |
| 7 | | 4 | | 0.32 | | 12 | | 0.47 | |  |  |  |  |  |  |
| 8 | | 0 | | 0.00 | | 8 | | 0.31 | |  |  |  |  |  |  |
| 9 | | 2 | | 0.16 | | 10 | | 0.39 | |  |  |  |  |  |  |
| 10 | | 1 | | 0.08 | | 8 | | 0.31 | |  |  |  |  |  |  |
| 11 | | 1 | | 0.08 | | 1 | | 0.04 | |  |  |  |  |  |  |
| 12 | | 0 | | 0.00 | | 3 | | 0.12 | |  |  |  |  |  |  |
| 13 | | 0 | | 0.00 | | 2 | | 0.08 | |  |  |  |  |  |  |
| 14 | | 0 | | 0.00 | | 3 | | 0.12 | |  |  |  |  |  |  |
| 15 | | 0 | | 0.00 | | 0 | | 0.00 | |  |  |  |  |  |  |
| 16 | | 0 | | 0.00 | | 0 | | 0.00 | |  |  |  |  |  |  |
| 17 | | 0 | | 0.00 | | 1 | | 0.04 | |  |  |  |  |  |  |
| **Screened following recruitment via both methods** | | 114 | | 9.25 | | 59 | | 2.30 | |  |  |  |  |  |  |
| Abbreviations: MSM=cisgender men who have sex with men; RDS=respondent-driven sampling; TW=transgender  women; VBS=venue-based sampling. | | | | | | | | | | | |  |  |  |  |

| **Supplemental Table 2. MSM and TW in Tijuana, Mexico tested and newly diagnosed with HIV infection following VBS recruitment by venue and venue type.** | | | | | |
| --- | --- | --- | --- | --- | --- |
| **Venue** | **# Tested** | **% Newly diagnosed**  **HIV-positive** | | | |
| **Virtual venue** | 9 | 11.1 | | | |
| 1 | 9 | 11.1 | | | |
| **Bar or club** | 586 | 6.5 | | | |
| 2 | 0 | 0.0 | | | |
| 3 | 1 | 0.0 | | | |
| 4 | 2 | 0.0 | | | |
| 5 | 5 | 0.0 | | | |
| 6 | 27 | 3.7 | | | |
| 7 | 0 | 0.0 | | | |
| 8 | 75 | 9.3 | | | |
| 9 | 172 | 5.8 | | | |
| 10 | 5 | 0.0 | | | |
| 11 | 130 | 8.5 | | | |
| 12 | 102 | 5.9 | | | |
| 13 | 20 | 10.0 | | | |
| 14 | 47 | 2.1 | | | |
| **Bathhouse** | 101 | 9.9 | | | |
| 15 | 57 | 14.0 | | | |
| 16 | 44 | 4.5 | | | |
| **Public space** | 380 | 5.5 | | | |
| 17 | 2 | 0.0 | | | |
| 18 | 51 | 13.7 | | | |
| 19 | 53 | 1.9 | | | |
| 20 | 0 | 0.0 | | | |
| 21 | 0 | 0.0 | | | |
| 22 | 215 | 3.7 | | | |
| 23 | 5 | 0.0 | | | |
| 24 | 4 | 25.0 | | | |
| 25 | 2 | 0.0 | | | |
| 26 | 2 | 0.0 | | | |
| 27 | 12 | 25.0 | | | |
| 28 | 34 | 2.9 | | | |
| **Other physical venue†** | 234 | 5.1 | | | |
| 29 | 73 | 1.4 | | | |
| 30 | 0 | 0.0 | | | |
| 31 | 0 | 0.0 | | | |
| 32 | 26 | 11.5 | | | |
| 33 | 2 | 0.0 | | | |
| 34 | 23 | 8.7 | | | |
| 35 | 110 | 5.5 | | | |
| **Special event** | 32 | 0.0 | | | |
| 36 | 2 | 0.0 | | | |
| 37 | 1 | 0.0 | | | |
| 38 | 16 | 0.0 | | | |
| 39 | 0 | 0.0 | | | |
| 40 | 0 | 0.0 | | | |
| 41 | 13 | 0.0 | | | |
| † Other physical venues include: hotel or motel, sex shop, adult movie theater, casino, and gym. | | |  |  |  |
| Abbreviations: MSM=cisgender men who have sex with men; TW=transgender women; VBS=venue-based sampling. | | | | |  |

| **Supplemental Table 3. Cross-group recruitment probabilities, homophily, and relevant proportions by age, gender identity, sexual orientation, sexual activity, and HIV status among MSM and TW recruited via RDS in Tijuana, Mexico.†‡** | | | | | |  |
| --- | --- | --- | --- | --- | --- | --- |
|  | Age of recruit | |  |  |  | |
| Age of recruiter | < 30 years | ≥ 30 years | Total |  |  | |
| < 30 years | 85 (60.3%) | 56 (39.7%) | 141 (100%) |  |  | |
| ≥ 30 years | 97 (12.2%) | 696 (87.8%) | 793 (100%) |  |  | |
| Total | 182 | 752 | 934 |  |  | |
| Sample proportion | 29.5% | 80.5% | 100% |  |  | |
| Equilibrium sample proportion | 23.5% | 76.5% | 100% |  |  | |
| Homophily | 0.49 | 0.45 | - |  |  | |
| Estimated mean network size | 4.47 | 4.11 | - |  |  | |
| Number of waves required to reach equilibrium | - | - | 5 |  |  | |
| Estimated population proportion (95% CI) | 22.1% (17.2-27.2) | 77.9% (72.8-82.8) | - |  |  | |
|  | Gender identity of recruit | |  |  |  | |
| Gender identity of recruiter | TGF | CM | Total |  |  | |
| Transgender female (TGF) | 3 (15.8%) | 16 (84.2%) | 19 (100%) |  |  | |
| Cisgender male (CM) | 14 (1.5%) | 901 (98.5%) | 915 (100%) |  |  | |
| Total | 17 | 917 | 934 |  |  | |
| Sample proportion | 1.8% | 98.2% | 100% |  |  | |
| Equilibrium sample proportion | 1.8% | 98.2% | 100% |  |  | |
| Homophily | 0.15 | -0.01 | - |  |  | |
| Estimated mean network size | 14.0 | 4.1 | - |  |  | |
| Number of waves required to reach equilibrium | - | - | 4 |  |  | |
| Estimated population proportion (95% CI) | 0.5% (0.2-0.9) | 99.5% (99.1-99.8) | - |  |  | |
|  | Sexual orientation of recruit | | | |  | |
| Sexual orientation of recruiter | Heterosexual | Bisexual | Gay | Other/not sure | Total | |
| Heterosexual | 41 (27.2%) | 90 (59.6%) | 15 (9.9%) | 5 (3.3%) | 151 (100%) | |
| Bisexual | 93 (17.9%) | 339 (65.3%) | 72 (13.9%) | 15 (2.9%) | 519 (100%) | |
| Gay | 20 (8.0%) | 81 (32.5%) | 144 (57.8%) | 4 (1.6%) | 249 (100%) | |
| Other/not sure | 6 (40.0%) | 7 (46.7%) | 2 (13.3%) | 0 (0.0%) | 15 (100%) | |
| Total | 160 | 517 | 233 | 24 | 934 | |
| Sample proportion | 17.1% | 55.4% | 24.9% | 2.6% | 100% | |
| Equilibrium sample proportion | 17.8% | 56.1% | 23.5% | 2.6% | 100% | |
| Homophily | 0.07 | 0.22 | 0.47 | -1.00 | - | |
| Estimated mean network size | 3.4 | 4.2 | 4.9 | 4.1 | - | |
| Number of waves required to reach equilibrium | - | - | - | - | 5 | |
| Estimated population proportion (95% CI) | 21.7% (17.6-26.5) | 55.7% (50.2-60.8) | 20.0% (15.7-24.9) | 2.6% (1.4-4.2) | - | |
|  | Sexual activity of recruit | |  |  |  | |
| Sexual activity of recruiter | No anal sex in the past 4 months | Anal sex  past 4 months | Total |  |  | |
| No anal sex with the past 4 months | 22 (18.8%) | 95 (81.2%) | 117 (100%) |  |  | |
| Anal sex in the past 4 months | 113 (13.8%) | 704 (86.2%) | 817 (100%) |  |  | |
| Total | 135 | 799 | 934 |  |  | |
| Sample proportion | 14.5% | 85.5% | 100% |  |  | |
| Equilibrium sample proportion | 14.6% | 85.4% | 100% |  |  | |
| Homophily | -0.06 | 0.31 | - |  |  | |
| Estimated mean network size | 3.0 | 4.4 | - |  |  | |
| Number of waves required to reach equilibrium | - | - | 1 |  |  | |
| Estimated population proportion (95% CI) | 20.1% (16.4-24.1) | 79.9% (75.9-83.6) | - |  |  | |
|  | HIV status of recruit | | |  |  | |
| HIV status of recruiter | HIV-negative | Newly diagnosed HIV-positive | Previously diagnosed HIV-positive | Total |  | |
| HIV-negative | 823 (93.6%) | 34 (3.9%) | 22 (2.5%) | 879 (100%) |  | |
| Newly diagnosed HIV-positive | 26 (78.8%) | 5 (15.2%) | 2 (6.1%) | 33 (100%) |  | |
| Previously diagnosed HIV-positive | 13 (59.1%) | 4 (18.2%) | 5 (22.7%) | 22 (100%) |  | |
| Total | 862 | 43 | 29 | 934 |  | |
| Sample proportion | 92.3% | 4.6% | 3.1% | 100% |  | |
| Equilibrium sample proportion | 91.7% | 4.9% | 3.4% | 100% |  | |
| Homophily | 0.26 | 0.09 | 0.21 | - |  | |
| Estimated mean network size | 4.2 | 3.1 | 7.1 | - |  | |
| Number of waves required to reach equilibrium | - | - | - | 2 |  | |
| Estimated population proportion (95% CI) | 91.4% (88.1-94.2) | 6.6% (4.0-9.5) | 2.0% (1.1-3.5) | - |  | |

† RDS Analysis Tool (Version 7.1.46) was used to estimate homophily, equilibrium proportions, and population estimates for the RDS sample.

‡ While there was evidence of weak to moderate homophily with respect to age, gender identity, sexual orientation, sexual activity, and HIV status, the average number of waves exceeded that needed to reach equilibrium (i.e., stable sample composition independent of the seeds) with respect to these factors and their distribution in the sample approximated that estimated under equilibrium.

Abbreviations: CI=confidence interval; MSM=cisgender men who have sex with men; RDS=respondent-driven sampling; TW=transgender women.

| **Supplemental Table 4. Characteristics of MSM and TW in Tijuana, Mexico newly diagnosed with HIV infection following recruitment via RDS by VBS recruitment venue attendance in the past 4 months.** | | | | | | |  |  |  |  |  |  |  |
| --- | --- | --- | --- | --- | --- | --- | --- | --- | --- | --- | --- | --- | --- |
|  | **Did not attend VBS venues (N=19)** | | **Attended VBS venues (N=12)** | |  |  |  |  |  |  |  |  |  |
|  | **n** | **%** | **n** | **%** | | **p-value** | |  |  |  |  |  |  |
| **Socio-demographics** |  |  |  |  | |  | |  |  |  |  |  |  |
| Median age (years) | 38.0 | IQR=28.0-49.0 | 41.0 | IQR=30.5-45.0 | | 0.73 | |  |  |  |  |  |  |
| Age (years) |  |  |  |  | | 0.18 | |  |  |  |  |  |  |
| 18 - 24 | 4 | 21.1 | 2 | 16.7 | |  | |  |  |  |  |  |  |
| 25 - 29 | 1 | 5.3 | 1 | 8.3 | |  | |  |  |  |  |  |  |
| 30 - 39 | 6 | 31.6 | 2 | 16.7 | |  | |  |  |  |  |  |  |
| 40 - 49 | 4 | 21.1 | 7 | 58.3 | |  | |  |  |  |  |  |  |
| 50+ | 4 | 21.1 | 0 | 0.0 | |  | |  |  |  |  |  |  |
| Gender identity |  |  |  |  | | 0.39 | |  |  |  |  |  |  |
| Cisgender male | 19 | 100.0 | 11 | 91.7 | |  | |  |  |  |  |  |  |
| Transgender female | 0 | 0.0 | 1 | 8.3 | |  | |  |  |  |  |  |  |
| Median years of residence in Tijuana | 8.0 | IQR=1.0-15.0 | 5.5 | IQR=2.5-8.5 | | 0.48 | |  |  |  |  |  |  |
| Sexual orientation |  |  |  |  | |  | |  |  |  |  |  |  |
| Heterosexual | 2 | 10.5 | 0 | 0.0 | | 0.83 | |  |  |  |  |  |  |
| Bisexual | 7 | 36.8 | 5 | 41.7 | |  | |  |  |  |  |  |  |
| Gay | 9 | 47.4 | 7 | 58.3 | |  | |  |  |  |  |  |  |
| Other/not sure | 1 | 5.3 | 0 | 0.0 | |  | |  |  |  |  |  |  |
| ≥ High school education | 12 | 63.2 | 4 | 33.3 | | 0.15 | |  |  |  |  |  |  |
| Employed | 7 | 36.8 | 9 | 75.0 | | 0.07 | |  |  |  |  |  |  |
| Average monthly income ≥$3,000 MXN (~$150 USD) | 0 | 0.0 | 0 | 0.0 | | - | |  |  |  |  |  |  |
| History of incarceration | 3 | 15.8 | 4 | 33.3 | | 0.38 | |  |  |  |  |  |  |
| History of deportation from the US | 6 | 33.3 | 6 | 50.0 | | 0.46 | |  |  |  |  |  |  |
| **Psychosocial factors** |  |  |  |  | |  | |  |  |  |  |  |  |
| Social support, median score (range: 0-100) | 53.1 | IQR=37.5-96.9 | 75.0 | IQR=57.8-95.3 | | 0.33 | |  |  |  |  |  |  |
| Internalized stigma, median score (range: 9-45) | 26.0 | IQR=22.0-32.0 | 25.0 | IQR=22.0-35.0 | | 0.31 | |  |  |  |  |  |  |
| Outness, median score (range: 1-7) | 4.0 | IQR=1.0-7.0 | 6.0 | IQR=2.5-7.0 | | 0.17 | |  |  |  |  |  |  |
| Depressive symptoms (CESD-10 score ≥10) | 8 | 42.1 | 6 | 50.0 | | 0.72 | |  |  |  |  |  |  |
| **Substance use behaviors** |  |  |  |  | |  | |  |  |  |  |  |  |
| Any illicit drug use (past month)† | 6 | 31.6 | 7 | 58.3 | | 0.26 | |  |  |  |  |  |  |
| Any injection drug use (past month) | 2 | 10.5 | 4 | 33.3 | | 0.17 | |  |  |  |  |  |  |
| Hazardous alcohol consumption (AUDIT score ≥7) | 3 | 15.8 | 3 | 25.0 | | 0.65 | |  |  |  |  |  |  |
| **Sexual behaviors (past 4 months)** |  |  |  |  | |  | |  |  |  |  |  |  |
| Any alcohol or illicit drug use before or during sex† | 13 | 68.4 | 11 | 91.7 | | 0.20 | |  |  |  |  |  |  |
| Any condomless anal sex | 16 | 84.2 | 9 | 75.0 | | 0.65 | |  |  |  |  |  |  |
| Any exchange of something of value for sex | 5 | 26.3 | 5 | 41.7 | | 0.45 | |  |  |  |  |  |  |
| **Sexual network characteristics (past 4 months)** |  |  |  |  | |  | |  |  |  |  |  |  |
| Median sexual network size | 2.0 | IQR=2.0-7.0 | 6.0 | IQR=5.0-10.0 | | 0.41 | |  |  |  |  |  |  |
| Any HIV-positive/status unknown sexual partners | 16 | 84.2 | 11 | 91.7 | | 1.00 | |  |  |  |  |  |  |
| Any main or primary sexual partners | 6 | 31.6 | 2 | 16.7 | | 0.43 | |  |  |  |  |  |  |
| Any cisgender female sexual partners | 7 | 36.8 | 2 | 16.7 | | 0.42 | |  |  |  |  |  |  |
| Any transgender female sexual partners | 2 | 10.5 | 2 | 16.7 | | 0.63 | |  |  |  |  |  |  |
| **HIV knowledge and prevention** |  |  |  |  | |  | |  |  |  |  |  |  |
| HIV knowledge, median score (range: 0-18) | 16.0 | IQR=15.0-16.0 | 16.0 | IQR=14.5-17.5 | | 0.87 | |  |  |  |  |  |  |
| Lifetime HIV testing‡ | 10 | 71.4 | 6 | 66.7 | | 1.00 | |  |  |  |  |  |  |
| HIV testing (past 12 months)‡ | 5 | 35.7 | 4 | 44.4 | | 1.00 | |  |  |  |  |  |  |
| †Illicit drugs include methamphetamine, cocaine, heroin, inhalants, amyl nitrites (poppers), ecstasy, gamma-hydroxybutyrate, ketamine, tranquilizers, or barbiturates. | | | | | | | | | |  |  |  |  |
| ‡Collected at 2-week follow-up visit, data missing for 11 participants who did not return for that visit. | | | | | | | | |  |  |  |  |  |
| Abbreviations: AUDIT=Alcohol Use Disorders Identification Test; CESD-10=10-item Center for Epidemiologic Studies Depression Scale; IQR=interquartile range;  MSM=cisgender men who have sex with men; MXN=Mexican pesos; RDS=respondent-driven sampling; TW=transgender women; USD=United States dollar; VBS=venue-based sampling. | | | | | | | | | | | | | |
| Numbers may not sum to column total due to missing data; percentages may not sum to 100 due to rounding or omission of one category for binary variables. | | | | | | | | | |  |  |  |  |
